# Melatonin: The Multifaceted Molecule in Plant Growth and Defense

**DOI:** 10.3390/ijms25126799

**Published:** 2024-06-20

**Authors:** Murtaza Khan, Adil Hussain, Byung-Wook Yun, Bong-Gyu Mun

**Affiliations:** 1 Department of Horticulture and Life Science, Yeungnam University, Gyeongsan 38541, Republic of Korea; 2 Department of Applied Biosciences, Kyungpook National University, Daegu 41566, Republic of Korea; 3 Department of Entomology, Abdul Wali Khan University Mardan, Mardan 23200, Pakistan; 4 Department of Environmental and Biological Chemistry, Chungbuk National University, Cheongju 28644, Republic of Korea

**Keywords:** melatonin, biosynthesis, plant growth, abiotic stress, biotic stress

## Abstract

Melatonin (MEL), a hormone primarily known for its role in regulating sleep and circadian rhythms in animals, has emerged as a multifaceted molecule in plants. Recent research has shed light on its diverse functions in plant growth and defense mechanisms. This review explores the intricate roles of MEL in plant growth and defense responses. MEL is involved in plant growth owing to its influence on hormone regulation. MEL promotes root elongation and lateral root formation and enhances photosynthesis, thereby promoting overall plant growth and productivity. Additionally, MEL is implicated in regulating the circadian rhythm of plants, affecting key physiological processes that influence plant growth patterns. MEL also exhibits antioxidant properties and scavenges reactive oxygen species, thereby mitigating oxidative stress. Furthermore, it activates defense pathways against various biotic stressors. MEL also enhances the production of secondary metabolites that contribute to plant resistance against environmental changes. MEL’s ability to modulate plant response to abiotic stresses has also been extensively studied. It regulates stomatal closure, conserves water, and enhances stress tolerance by activating stress-responsive genes and modulating signaling pathways. Moreover, MEL and nitric oxide cooperate in stress responses, antioxidant defense, and plant growth. Understanding the mechanisms underlying MEL’s actions in plants will provide new insights into the development of innovative strategies for enhancing crop productivity, improving stress tolerance, and combating plant diseases. Further research in this area will deepen our knowledge of MEL’s intricate functions and its potential applications in sustainable agriculture.

## 1. Introduction

Melatonin (MEL), a hormone best known for its role in regulating sleep and circadian rhythms in animals, has emerged as a multifaceted molecule with diverse functions in plants [1]. In addition to animals, MEL has been discovered in various plant species, where it influences plant growth and defense mechanisms [2]. This multifaceted molecule has attracted substantial attention from researchers worldwide, as its involvement in plant physiology opens up exciting possibilities for understanding and manipulating key processes in plant development and protection [3].

Numerous studies have elucidated the intricate roles of MEL in different aspects of plant physiology, including seed germination, root development, shoot growth, flowering, fruit ripening, and stress responses [4,5]. Moreover, the impact of MEL on the regulation of hormones in plants, such as auxins (AUXs), gibberellins (GAs), abscisic acid (ABA), and cytokinins (CKs), has been investigated, revealing its ability to modulate these signaling pathways and consequently influence various stages of plant growth [6,7].

Furthermore, MEL has been found to play a crucial role in plant defense mechanisms [8]. MEL is recognized as a critical component of plant defense responses against abiotic stressors [9]. Under abiotic stress conditions, MEL levels often increase in plant tissues [10]. MEL acts as a potent antioxidant, protecting plants from oxidative damage caused by reactive oxygen species (ROS) [11]. It scavenges ROS and maintains redox homeostasis in cells [12]. MEL is involved in regulating the expression of abiotic stress-responsive genes and modulating signaling pathways related to stress tolerance, thereby allowing plants to adapt and survive under challenging environmental conditions [13]. In terms of biotic stress, MEL participates in plant defense against pathogens and pests [14]. It also contributes to the synthesis of defense-related compounds, such as phytoalexins, pathogenesis-related proteins, and secondary metabolites with antimicrobial properties [15]. MEL interacts with plant defense hormones, such as salicylic acid (SA), jasmonic acid (JA), and ethylene (ETH), to regulate the immune response and enhance plant resistance against biotic stressors [16].

MEL has also been implicated in regulating the circadian rhythm of plants [17]. By modulating key physiological processes, MEL affects growth patterns and synchronizes plant responses to various environmental cues [18]. MEL and nitric oxide (NO) are signaling molecules in plants that influence physiological processes, such as MEL synthesis and NO production. They cooperate in plant growth and defense against abiotic and biotic stressors [19].

Understanding the multifaceted role of MEL in plant growth and defense is crucial for agriculture and crop productivity [20]. Manipulating MEL levels or its signaling pathways holds promise for enhancing plant growth, improving stress tolerance, and combating plant diseases [16,21]. Consequently, exploring the mechanisms underlying MEL’s actions in plants can provide valuable insights into developing innovative strategies for sustainable agriculture.

In this review, we explore the multifaceted functions of MEL in plant growth and defense, highlighting its influence on hormone regulation, role in stress responses, and impact on various stages of plant development. By examining the latest research findings, we aim to shed light on the potential applications of MEL in agriculture practices and provide a basis for further investigation into the effects of MEL on plant physiology.

## 2. MEL Biosynthesis

**Figure 1:**
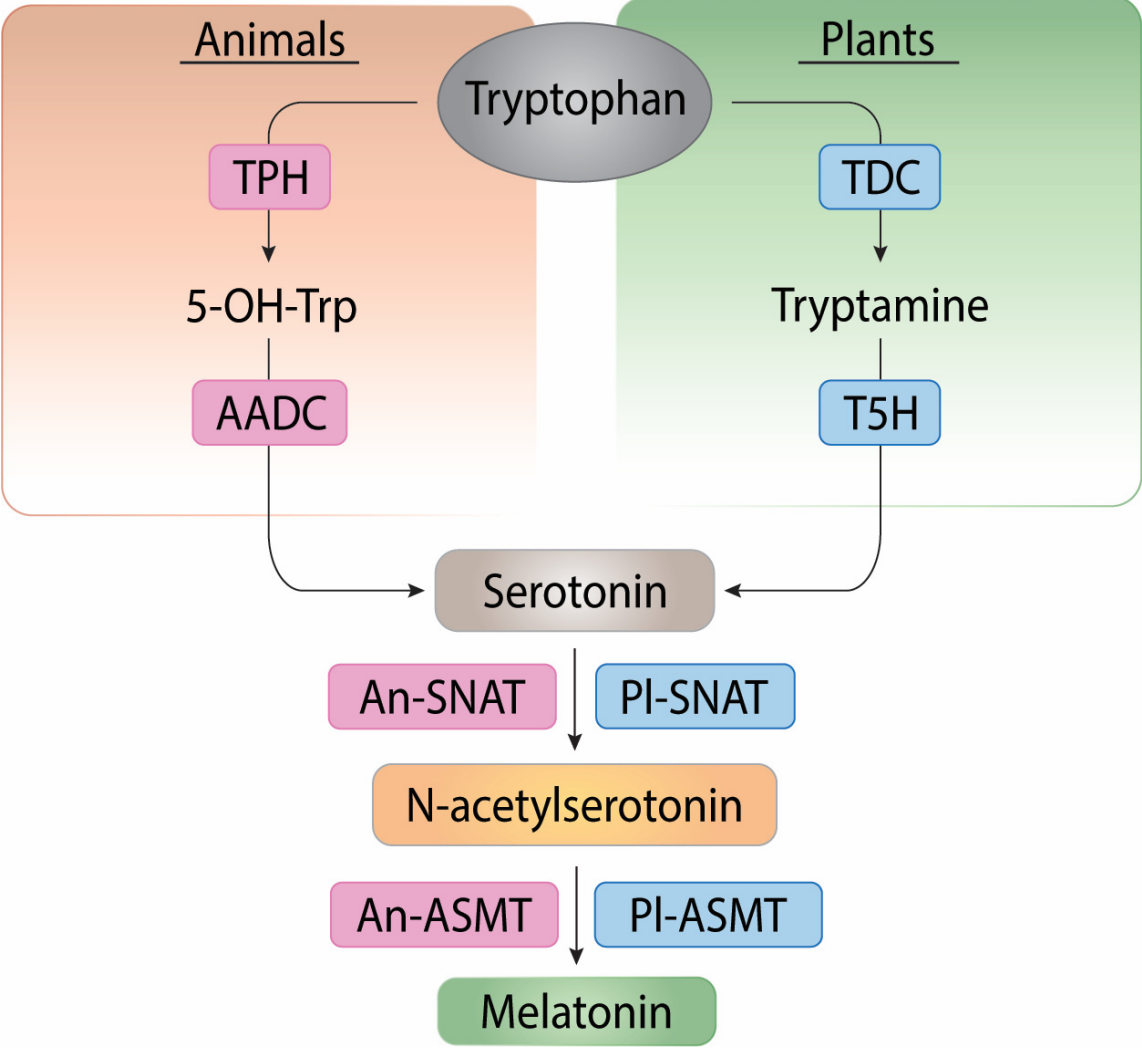
Biosynthesis of melatonin in animals and plants. Blue indicates the common products and pathways in plants and animals. Pink indicates the animal pathway. Green indicates the plant pathway.

MEL is biosynthesized in plants and animals through four enzymes: tryptophan hydroxylase (TPH), aromatic L-amino acid decarboxylase (AADC), serotonin N-acetyltransferase (SNAT)/alkylamine N-acetyltransferase (AANAT), and N-acetylserotonin O-methyltransferase (ASMT). In the first step, in animals, tryptophan is converted into tryptamine 5-hydroxylase (5-OH-Trp) by the action of TPH, whereas in plants, tryptophan is converted into tryptamine by the action of tryptophan decarboxylase (TDC). In the second step, in animals, 5-OH-Trp is converted into serotonin by the action of AADC, whereas in plants, tryptamine is converted into serotonin by the action of tryptamine 5-hydroxylase. In the third step, in animals, serotonin is converted into N-acetylserotonin by the action of Animal-SNAT (An-SNAT), whereas in plants, serotonin is converted into N-acetylserotonin by the action of Plant-SNAT (Pl-SNAT). In the fourth and final step, in animals, N-acetylserotonin is converted into MEL by the action of Animal-ASMT, whereas in plants, N-acetylserotonin is converted into MEL by the action of Plant-ASMT [22]. The biosynthesis of MEL from tryptophan in plants and animals is shown in Figure 1.

## 3. Role of MEL in Plant Growth and Development

MEL was first discovered in plants by Dubbels et al. [23] and Hattori et al. [24]. Since then, several authors have reported that MEL plays a significant role in seed germination, the root system of plants, circadian rhythms, photosynthesis, flowering, senescence, and adaptation to biotic and abiotic stresses [2,25,26,27,28,29]. Recent investigations have suggested that MEL plays a role as a new phytohormone or growth regulator in plants, and the structural similarities between indole-3-acetic acid (IAA, AUX) and MEL have attracted plant scientists to investigate its role in the growth and development of plants [30,31].

### 3.1. Role of MEL in the Vegetative Growth of Plants

Seed germination is the process by which a seed develops into a new plant [32]. Zhang et al. [33] and Xiao et al. [34] reported that MEL modulates cell division, shoot initiation, and seed dormancy of *Arabidopsis*, cucumber, bermudagrass, pepper, sweet corn, and cotton. To accelerate seed germination, MEL interacts with phytohormones, such as IAA, ABA, GA, and CKs [35,36]. At optimum/low concentrations (20 µM), MEL increased the seed germination of cotton, but at higher concentrations (50, 100, and 200 µM), it reduced and even inhibited the seed germination [34]. Chen et al. [37] reported that 20 µM of MEL was more effective in increasing the germination of cotton seeds. In contrast, Lv et al. [38] reported that MEL did not induce seed germination in *Arabidopsis thaliana* at low concentrations (10 and 100 µM) and inhibited it at higher concentrations (500 and 1000 µM). However, MEL has been reported to accelerate seed germination in different plants, thereby improving their productivity and survival [39,40]. Further studies are needed to optimize the concentration of MEL for improving seed germination in different plant species.

Studies have shown that MEL significantly contributes to the regulation of root development in plants [41,42]. MEL application has been reported to improve the root system of several plants, such as rice, tomato, and apple [42,43]. The correlation of MEL with hormones such as IAA, ABA, GA, and zeatin riboside plays a crucial role in the development of adventitious roots [42]. However, the crosstalk is complex and MEL inhibits taproot growth in canary grass and oat regardless of its concentration [5]. In addition, the role of MEL in root development has led to the identification of novel signaling networks, such as hydrogen peroxide (H_2_O_2_) and NO [43,44]. These studies suggest that endogenous and exogenous MEL play a significant role in the development of the root system of plants to increase their growth, productivity, and adaptation to harsh environments. Further studies are needed to use MEL on a large scale to improve plant growth and survival.

MEL has been shown to considerably improve plant height, fresh and dry weight, leaf length and width, branching number, stem diameter, relative water content, chlorophyll content, net photosynthetic rate, stomatal conductance, maximum photochemical efficiency, photosystem II effective photon yield, photochemical quenching, and sugar content [5,40,45,46,47]. MEL also regulates the content of minerals in plants, such as phosphorous, potassium, calcium, zinc, copper, and manganese [48,49]. MEL regulates the growth of plants by regulating nitrogen (N) metabolism. Qiao et al. [50] reported that applying 1 µM of MEL in a hydroponic solution enhanced wheat seedling growth under N-sufficient and N-deficient conditions. Overall, these findings suggest that MEL enhances plant growth by regulating the physical, biochemical, and molecular aspects of plants and that MEL can be used as an important growth regulator of plants.

MEL is a circadian oscillator that influences the rhythm of the biological clock system and several physiological markers in humans and plants [17]. MEL levels are generally higher at night in most plants, including *Arabidopsis*, rice, and barley [17,18]. MEL synthesis is light-dependent and has regulatory interactions with photoreceptors, whose absence affects the expression of genes involved in MEL biosynthesis [18]. MEL treatment restores the rhythmic expression of core circadian clock genes, whereas the absence of GIGANTEA genes results in the nonrhythmic expression of ASMT, suggesting a potential melatonin-mediated signaling network [17,18].

MEL also has a pivotal role in the establishment of a symbiotic association with microorganisms to improve plant growth, productivity, and survival. Zhang et al. [51] reported that the application of MEL enhances the colonization of arbuscular mycorrhiza (AM) in *Medicago truncatula* plants. They also stated that AM plants under lead stress exhibited higher MEL levels than non-AM plants. They reported that MEL treatment enhanced plant growth by improving AM symbiosis, stimulating the antioxidant system, and reducing lead uptake. Imran et al. [52] and Alinia et al. [53] also reported that the synergistic application of *Lysinibacillus fusiformis* L. (PLT16) and rhizobacterium strain ameliorates the negative effects of drought and salt stress on soybean and bean plants by regulating photosynthesis and the hormonal and antioxidant systems. These findings indicate that MEL can enhance symbiotic association to accelerate plant growth and resistance to harsh environments.

**Figure 2:**
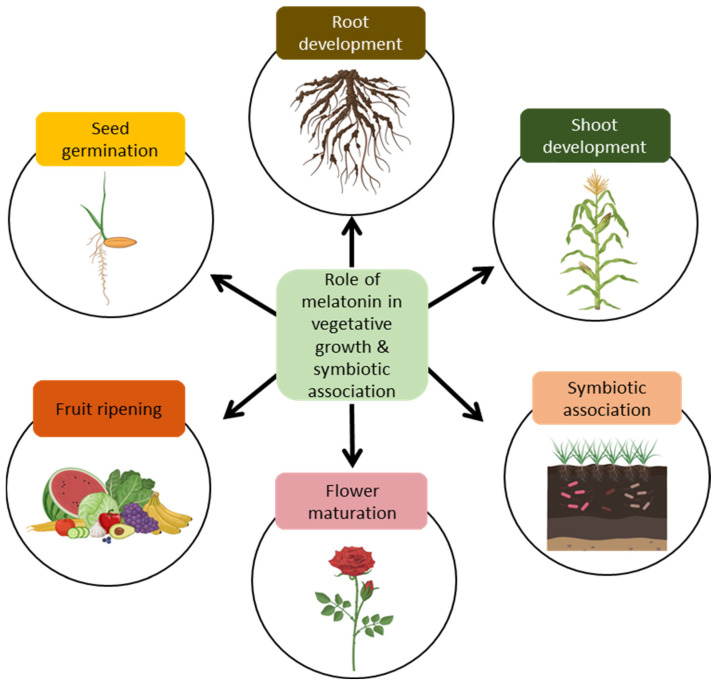
Role of melatonin in the growth and symbiotic association of plants.

The role of MEL in seed germination, root and shoot development, and symbiotic association with microorganisms to improve overall plant growth, productivity, and survival is shown in Figure 2.

### 3.2. Role of MEL in the Reproductive Growth of Plants

MEL has multiple functions in flowering, including activating Flowering Locus C, stabilizing transcriptional regulator DELLA proteins, and regulating volatile compound content [5,54]. It also reverses the inhibitory effect of stress on pollen viability and germination, promotes the transport of carbon from leaves to sink tissues, and maintains carbohydrate metabolism in male and female tissues [44]. In addition, it contributes to higher male fertility of crops by increasing autophagy-related gene expression and autophagosome formation [55]. Finally, it regulates the tricarboxylic acid cycle to meet energy demands under negative environmental conditions [56]. These studies indicate that MEL has the potential to regulate the reproductive growth of plants and can be used as a growth regulator and stress mitigator in plants.

### 3.3. Role of MEL in Fruit Maturation and Post-Management

MEL plays an important role in fruit ripening, production, and quality [57]. It increases the contents of organic acids, phenolics, flavonoids, peonidin derivatives, and apoptotic inhibitor proteins as well as influencing the expression of sucrose invertase genes and the net photosynthetic rate [58,59]. MEL also triggers the metabolism of most hormones; increases ABA, H_2_O_2_, and ethylene (ETH) content; participates in signaling molecules; and coordinates biochemical and developmental pathways that affect the texture and nutritional quality of fruits [59]. Fruits treated with MEL have higher quality, number, weight, and size, suggesting its role in regulating phytohormone synthesis [60,61].

MEL also maintains good storability and quality of fruits [59]. Exogenous application of MEL reduces ETH production during postharvest, delays senescence in cold-stored mangoes and tomatoes, and reduces water runoff to regulate ETH release [60,61,62,63]. MEL-based coatings influence the expression of several genes, such as wax synthesis genes (*CER1*), cutin monomer genes (*GPAT4/8*), and aquaporin genes (*PIP1;4*, *PIP2;7*, and *PIP22*), which all determine the formation of a surface barrier composed of a cuticle to reduce water outflow [64,65]. In addition, MEL benefits metabolic processes (respiration and transpiration), which help reduce the water vapor pressure gradient between the fruit and the surrounding atmosphere [66]. Fruits treated with MEL, such as mango and guava, can maintain significantly higher unsaturated fatty acid levels and higher activities of enzymes (cytochrome c oxidase, H-ATPase, and Ca-ATPase) than untreated fruits [63,67].

## 4. Role of MEL in the Mitigation of Abiotic Stresses

**Figure 3:**
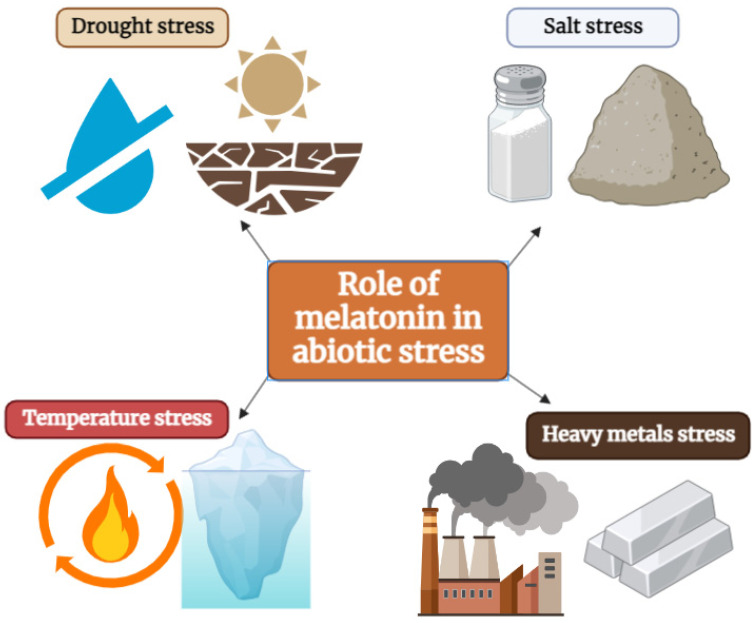
Role of melatonin in the mitigation of drought, salinity, temperature, and heavy metal stresses.

MEL acts as a multifunctional molecule in plants and aids in the mitigation of abiotic stresses as shown in Figure 3 by regulating the root system, stomatal closure, gene expression, photosynthesis, hormonal production, antioxidant system, osmotic balance, symbiotic association, and overall plant growth [4]. Its role in these processes makes it a valuable tool for improving plant resilience and productivity under normal and stressed environmental conditions.

### 4.1. Melatonin: A Drought Stress Mitigator

MEL has a significant impact on mitigating the adverse effects of drought stress on plants. Stomata are small pores on the surface of leaves that regulate gas exchange and water loss in plants. Under drought conditions, MEL enhances stomatal closure by reducing the rate of transpiration and water from the plants, which helps plants conserve water during drought stress [68]. Drought stress often leads to the production of ROS in plants, which can cause oxidative damage. However, MEL acts as an important regulator of the antioxidant system to scavenge excessive ROS and reduce the oxidative damage of drought stress on plants [69]. Plants increase the expression of drought-stress-responsive genes to tolerate drought stress. As a drought mitigator, MEL regulates the expression of *NCED3*, *ASMAP1*, and *ASPK11* [45,70]. Drought stress due to excessive transpiration disrupts the water balance and osmotic potential of plants. However, to protect the plants from drought stress, MEL adjusts the osmotic potential and water balance through the accumulation of proline, sugars, and polyamines [71,72,73]. Phytohormones, such as ABA and CKs, play a significant role in the induction of drought stress tolerance in plants. As a drought stress mitigator, MEL modulates the production of these hormones to enhance drought stress tolerance in plants [74]. MEL treatment has been reported to increase drought stress tolerance in several plants, such as rice, apple, kiwifruit, tobacco, tomato, and cucumber [75,76,77,78]. Further studies are needed to fully understand the mechanism of action and potential applications of MEL on a large scale in drought-prone areas.

### 4.2. Melatonin: A Salinity Stress Alleviator

MEL has also been reported to alleviate salinity stress in plants. Several studies have shown that MEL alleviates the adverse effects of salt stress to improve plant production and survival. In plants, osmotic adjustment is essential for maintaining water balance, protecting plant cells, and increasing salt stress tolerance and nutrient uptake. MEL plays a significant role in osmotic adjustment to maintain cellular hydration and protein stabilization as well as protect plant organs from the adverse effects of salt stress [37,79]. During salt stress, plants often experience oxidative stress due to over-produced ROS. However, plants regulate the production of ROS through a well-established antioxidant system [80]. MEL acts as a free radical scavenger to reduce excessive ROS accumulation and protect plants from oxidative damage during salt stress [81,82]. Ion homeostasis in plants refers to the balance and regulation of ion concentrations within plant cells and tissues. During salt stress, in which the soil contains high levels of salt (usually sodium chloride), ion homeostasis is disturbed, which leads to alterations in ion distribution and accumulation. MEL, as a salt stress ameliorator, regulates ion homeostasis through the uptake and transport of ions to prevent their toxic build-up in cells and tissues [79,83]. Conversely, it enhances the retention of essential ions, such as potassium (P^+^) and calcium (Ca^2+^), which are vital for plant growth and salt stress tolerance [84,85]. Salinity stress affects stomatal behavior, resulting in increased transpiration and water loss. MEL has been reported to regulate stomatal aperture, thereby promoting stomatal closure under salt stress conditions. This reduces water loss through transpiration and helps plants conserve water and maintain better water use efficiency [86]. Salt stress disrupts the hormonal balance in plants, including the synthesis and response of plant growth regulators. MEL regulates hormone signaling pathways, particularly the ABA pathway, which plays an important role in plant response to salt stress [46,87]. Salinity stress negatively affects root growth and morphology, limiting water and nutrient uptake. MEL improves the root system under salt stress by stimulating root elongation, increasing lateral root branching, and enhancing root hair formation, thereby improving the plant’s ability to explore the soil for water uptake [88,89]. The effectiveness of MEL in alleviating salt stress may vary among different plant species and genotypes. In addition, the optimal concentration and timing of MEL application need to be determined for specific plant species and growth conditions. Nevertheless, research on MEL’s role in enhancing salt tolerance is promising and it represents a potential avenue for developing strategies to mitigate salinity stress in crops and improve their productivity in salt-affected environments.

### 4.3. MEL Relieves Plants from Temperature Stress

Temperature stress includes heat and cold stress, and MEL has been reported as a potential chemical in mitigating the negative effects of these stressors [90,91]. High temperatures often lead to oxidative stress and cause oxidative damage to plants. MEL acts as a vital protectant for plants against heat stress by enhancing the antioxidant system and reducing ROS production [92]. Heat stress disrupts membrane integrity, leading to the leakage of cellular contents. MEL helps stabilize cellular membranes by preventing lipid peroxidation and maintaining membrane fluidity, thus protecting the structural and functional integrity of cells [93,94]. MEL also regulates the expression of genes and the production of heat-shock proteins and hormones that are involved in heat stress responses [6,95,96].

Cold stress, which includes exposure to low temperatures, adversely affects plant growth and development. However, MEL as a chemical protector significantly enhances cold stress tolerance in plants [97]. Cold stress impairs photosynthesis in plants, leading to reduced growth and productivity. MEL, as a cold stress mitigator, significantly improves photosynthetic efficiency under cold stress conditions by enhancing the activities of key enzymes involved in carbon fixation, such as ribulose-1,5-biphosphate carboxylase/oxygenase (RUBISCO) [98,99]. Cold stress disrupts the integrity and fluidity of cell membranes, leading to cellular damage. MEL improves the stability and fluidity of cell membranes, thereby protecting them from the adverse effects of low temperatures [100]. MEL also increases the expression of genes involved in cold stress tolerance to enhance the tolerance of plants [101]. To further enhance cold stress tolerance in plants, MEL interacts with phytohormones and increases the activity of antioxidants [97,102]. The antioxidant properties of MEL and its ability to stabilize the plant membrane, regulate gene expression, enhance photosynthesis, and modulate hormonal balance collectively contribute to improving plant growth and survival under cold stress conditions. However, the precise mechanism of MEL action in plants, including its role in cold stress tolerance, is still under investigation, and further studies are needed to fully understand the functions and potential applications of MEL in agriculture.

### 4.4. Melatonin: A Protectant from Heavy Metal (HM) Stress

In addition to its role in abiotic stress tolerance, MEL has been found to act as a protectant against heavy metal (HM) stress in plants. HMs, such as lead, mercury, cadmium, and arsenic, accumulate in the soil and pose a significant threat to plant growth and development [103,104]. However, MEL protects plants from the toxic effects of HMs through chelation [105]. Chelation allows MEL to reduce the bioavailability and mobility of HMs, preventing their uptake and accumulation in plant tissues. By forming stable complexes with HMs, MEL helps detoxify and sequester these toxic elements, reducing their damaging effects on plants [106]. Phytochelatins (PCs) are small peptides that play a critical role in HM detoxification by chelating HMs and sequestering them in vacuoles. MEL stimulates the synthesis of PCs in plants exposed to HMs. By promoting PC production, MEL enhances the ability of plants to bind and detoxify HMs [107]. HMs induce oxidative stress in plants by promoting the generation of ROS. MEL, as a potent antioxidant, scavenges ROS and minimizes oxidative damage to plant cells [108]. Furthermore, MEL enhances the activities of antioxidant enzymes, such as SOD, CAT, and POD, under HM stress [109]. HMs trigger changes in gene expression patterns in plants. MEL plays a significant role in the regulation of the expression of genes involved in HM detoxification and tolerance mechanisms. MEL upregulates the expression of metallothionein, which are small, metal-binding proteins that sequester HMs and protect against their toxicity [110]. MEL also modulates the expression of transporters involved in the exclusion or compartmentalization of HMs within plants [111]. The protective effects of MEL against HMs in plants make it a promising candidate for phytoremediation strategies, where plants are used to remove HMs from contaminated soils [112]. Moreover, the exogenous application of MEL has been shown to alleviate HM toxicity in plants, offering a potential approach to enhance plant resilience in HM-contaminated environments [113]. While MEL shows potential in mitigating HM stress in plants, further research is needed to fully understand its mechanisms of action and potential application in practical settings. Table 1 systematical outlines the role of MEL in abiotic stress and growth and development.

## 5. Role of MEL in the Mitigation of Biotic Stresses

Although the primary function of MEL in plants is related to regulating growth and development, there is increasing evidence that MEL also plays a role in plant defense against stressors, such as herbivores and pathogens [116].

### 5.1. Role of MEL in Resistance against Herbivores

MEL induces the production of various defense-related compounds in plants that are toxic or repellent to herbivores. For instance, MEL increases the production of secondary metabolites, such as phenolics, alkaloids, and terpenoids, which deter or harm herbivores upon ingestion or contact [117]. MEL modulates various signaling pathways involved in plant defense against herbivores [118]. Furthermore, MEL influences the levels and activities of plant hormones, such as JA and ETH, which play a pivotal role in the induction of defense against herbivores. By regulating these signaling pathways, MEL induces the ability of plants to detect and respond to herbivory [2]. MEL primes plant defense responses, making them more rapid and efficient upon herbivore attack [119]. MEL also increases the expression of genes related to herbivore resistance [120]. Herbivore behavior is influenced by MEL. It has been observed to impact the feeding and oviposition (egg-laying) habits of several herbivorous insects. MEL in plant tissues discourages herbivores or attracts their natural enemies, such as predators or parasitoids, thereby lowering herbivore populations [120,121]. MEL functions as an antioxidant in plants and reduces the oxidative damage caused by herbivory. ROS, which are produced by herbivore grazing, cause cellular damage in plant tissues. MEL acts against the detrimental effects of ROS through its antioxidant capability, improving plant health by lowering herbivory [122]. The specific effects of MEL on herbivore resistance may change depending on the plant species, herbivore types, and ecological situation. Although information about MEL’s function in plant defense against herbivores is still limited, MEL has been reported to be involved in plant resistance to herbivory through its effects on defense compounds, signaling pathways, defense priming, herbivore behavior modulation, and antioxidant activity.

### 5.2. Antiviral Effects of MEL

MEL, a plant growth regulator, also exhibits antiviral properties and protects plants from toxic effects. Viral infections negatively impact the photosynthetic system of plants, leading to reduced plant growth and productivity. MEL protects the photosynthetic system of plants from viruses through the reduction of oxidative stress [123,124]. MEL exhibits direct antiviral activity against certain plant viruses. MEL inhibits viral replication and reduces the spread of infection within plant tissues. This antiviral activity is attributed to the ability of MEL to induce the production of antiviral proteins and other defense-related molecules [125]. MEL triggers the activation of plant defense responses, including the expression of defense-related genes and the production of hormones and secondary metabolites. These responses strengthen the plant’s defense system against viral infections [6,126,127]. The exact mechanism through which MEL confers resistance against viral diseases in plants is still under investigation. Nonetheless, the emerging evidence suggests that MEL has potential as a plant protectant against viral infections and can be used for developing sustainable strategies for disease control in agriculture.

### 5.3. Antibacterial Effects of MEL

MEL has also been found to exhibit antibacterial effects. MEL is involved in the regulation of gene expression in plants. It enhances the expression of genes that encode antimicrobial peptides, pathogenesis-related proteins, and other defense-related molecules. These gene products play an important role in plant defense against bacterial pathogens [128]. MEL interacts with phytohormones, such as SA, JA, and ETH, to enhance plant immunity against bacterial infection by modulating the concentration and activities of these hormones [2,129]. MEL also exhibits direct antibacterial properties against certain plant bacterial pathogens and inhibits the growth and proliferation of bacteria by disrupting their physiology [130]. It also interferes with bacterial cell division, affects bacterial cell membrane integrity, and induces oxidative stress, ultimately leading to bacterial cell death [131]. MEL also induces plant immune responses against pathogenic bacteria. It enhances the innate immune system of plants, leading to the synthesis of ROS, cell wall strengthening, and induction of programmed cell death (PCD) in the infected parts of the plants. These responses limit bacterial infection and prevent further spread of the disease [132]. MEL also induces systemic acquired resistance (SAR) of plants against bacteria and other pathogenic organisms. Through activation of SAR in the uninfected parts of plants, MEL protects the plants from subsequent infections [133]. The antibacterial effects of MEL in plants have been demonstrated in several studies, highlighting its potential as a natural defense mechanism against bacterial pathogens. However, the efficiency of MEL as an antibacterial agent may vary depending on specific plant–pathogen interactions and environmental conditions. Further research is needed to fully understand the mechanisms underlying the antibacterial effects of MEL and its applications in plant protection.

### 5.4. Antifungal Effects of MEL

MEL also plays a significant role in the protection of plants from various fungal diseases. The antifungal effects of MEL in plants have been studied in several species, and the results suggest that MEL enhances plant resistance against fungal pathogens [134]. MEL triggers the production of defense-related compounds, such as phytoalexins, antimicrobial peptides, and pathogenesis-related proteins [135,136]. These compounds act as natural defense mechanisms and inhibit the growth and spread of fungal pathogens. MEL alters immunological signaling pathways in plants, thereby increasing their defenses against harmful fungi [118]. ROS are frequently produced in plant cells as a result of pathogenic fungal infections, which cause oxidative stress and damage. Strong antioxidant MEL scavenges ROS and reduces oxidative stress. MEL induces plants’ defenses against fungal infections by lowering oxidative damage [137]. PCD is an essential defense mechanism in plants against pathogenic fungi. MEL has been reported to regulate the balance between cell death and cell survival pathways, thereby improving the plant’s ability to eliminate infected cells while minimizing damage to healthy tissues. These regulated cell death processes contribute to the plant’s resistance against fungal infections [138]. MEL also plays a significant role in the induction of SAR to inhibit the further spread of fungal infections [139]. The specific mechanisms and effectiveness of MEL in conferring resistance to pathogenic fungi may vary among plant species and specific fungal pathogens. In addition, the application of exogenous MEL or genetic manipulation of MEL-related genes may have different effects on resistance depending on the experimental conditions and plant species. Further research is necessary to fully understand the intricate mechanisms underlying MEL-mediated resistance against pathogenic fungi and its potential application in crop protection strategies.

In rice, MEL improves resistance to stripe virus infection via a NO-dependent pathway, whereas in *Arabidopsis*, MEL and NO cooperate to upregulate the expression of multiple SA-related genes [126,140]. MAPK cascades and oxidative signal inducible 1 (OXI1) kinases are responsible for MEL-triggered innate immunity signal transduction [141]. Further investigations using genomic and proteomic approaches will help unravel the interactions of MEL with other components as well as the roles of SA, JA, ETH, NO, and MEL receptors in cascading immunity signals [126,142].

MEL is an alkaloid compound that has a bitter and unpleasant taste, which helps in protecting plants against herbivores [143]. Plants with high levels of MEL, such as walnut, if consumed by herbivores, can increase the level of MEL in their bodies and disturb their physiology [144]. The resistance of rice plants to herbivores, such as leaffolder and brown planthopper, was significantly enhanced via the production of JA, SA, MEL, flavonoids, and lignin [120]. Similarly, American elm’s resistance was enhanced against the feeding of beetles by increasing the production of MEL, serotonin, and JA [114]. These studies indicate that MEL can be used to increase the resistance of plants against herbivores. MEL enhances the resistance of animals and plants against pathogenic bacteria, such as Gram-positive, Gram-negative, and *Mycobacterium tuberculosis*, by activating the MAPK pathway, ROS, and RNS to reduce microbial growth and their damage [145]. The application of MEL significantly reduced tobacco mosaic virus (TMV) viral RNA and its concentration in infected *Nicotiana glutinosa* and tomato seedlings [146]. Similarly, the application of MEL reduced the infection of apple stem grooving virus in apple plants [147]. MEL promoted tomato fruit resistance to *Botrytis cinerea* in a plant–fungi interaction model by controlling the production of H_2_O_2_ and the JA signaling pathway [118]. In watermelon and other cucurbits, an increase in MEL accumulation increases resistance to foliar diseases, such as powdery mildew and soil-borne oomycetes, through alterations in the expression of genes linked to effector-triggered immunity and pathogen-associated molecular pattern immunity [148]. The role of MEL in defense against herbivores, bacteria, viruses, and fungi is presented in Table 2.

## 6. Interaction of MEL with NO

**Figure 4:**
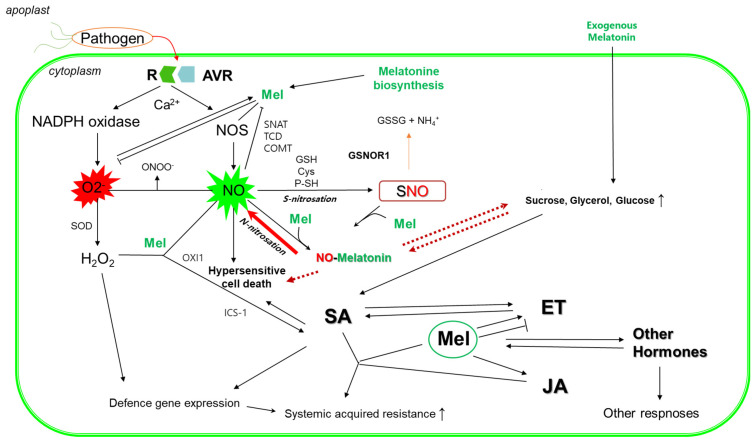
Role of melatonin in biotic interaction.

MEL and NO are signaling molecules that play important roles in various physiological processes in plants, including growth, development, and stress responses. While MEL and NO have distinct functions, they can interact and influence each other’s actions in plants. NO has been reported to regulate the synthesis of MEL in plants. Studies have shown that NO can enhance the expression of genes involved in MEL biosynthesis, such as *SNAT* and *ASMT* [19,153]. This suggests that NO can modulate MEL production in plants. MEL has also been found to stimulate the synthesis of NO in plants. It can increase the activity of enzymes involved in NO synthesis, such as nitrate reductase and nitric oxide synthase-like enzymes [19,115]. This indicates that MEL can promote NO production in plants, potentially leading to NO-mediated signaling events. MEL and NO are involved in plant responses to various abiotic and biotic stresses. They can act synergistically to enhance stress tolerance in plants. For example, MEL and NO have been shown to cooperate in the regulation of antioxidant systems, including the scavenging of ROS [19]. This suggests that MEL and NO act synersgistically to protect plants against oxidative stress. MEL and NO have been implicated in the regulation of seed germination. Studies have demonstrated that MEL can promote seed germination, and this effect is associated with increased NO production. Inhibition of NO synthesis can attenuate the stimulatory effect of MEL on seed germination, suggesting an interaction between the two molecules during this process. Overall, while the precise mechanisms of crosstalk between MEL and NO in plants are not fully understood, the available evidence suggests that they can influence each other’s synthesis and modulate similar physiological processes. Further research is needed to elucidate the specific molecular mechanisms underlying their interaction and functional consequences in plant biology. Melatonin’s involvement in plant defense is evident in its capacity to enhance resistance against herbivores, exhibit antibacterial and antifungal effects, and modulate signaling molecules such as nitric oxide (NO). Figure 4 provides a detailed visual representation of melatonin’s interactions with key signaling molecules, including jasmonic acid (JA), salicylic acid (SA), reactive oxygen species (ROS), and NO. 

## 7. Conclusions and Future Directions

MEL plays a crucial role in the growth and defense mechanisms of plants against biotic and abiotic stresses. It enhances plant growth by regulating various processes and interacting with plant growth regulators. MEL acts as an antioxidant and mitigates oxidative damage caused by stressors, such as drought, salinity, extreme temperatures, and HMs. It also increases the activities of antioxidant enzymes. Furthermore, MEL activates plant immune responses, including the production of defense compounds and proteins as well as modulation of gene expression. MEL and NO interact and influence the synthesis and actions of each other in various physiological processes. Overall, the diverse functions of MEL render it a promising candidate for improving plant performance and stress tolerance, although further research is needed to fully understand its mechanisms and applications.

## Figures and Tables

**Table 1:** Role of MEL in the Mitigation of Abiotic Stress and in Growth and Development.

Plants	Abiotic Stress/ Growth and Develpment	Mechanism	Method of Application	Dose	Effects	Ref.
Rice	Drought	ROS scavenging	Foliar spray	100 µM	Promoting signals to drought stress adaption	[75]
Apple	Drought	boosting in nutrient uptake.	Irrigation	100 µM	Enhancing plant tolerance and adaptability	[114]
Kiwifruit	Drought	Gene expression regulation	Seed priming	100 μM	Enhances seedling adaptability	[77]
Borage	Salt	Ion homeostasis, antioxidant system	Foliar application	100 µM	Improved ion regulation, reduced ROS damage	[79]
Tomato	Salt	Osmotic adjustment, hormonal signaling	Foliar spray	100 µM	Better water use efficiency, enhanced growth	[87]
Cucumber	Salt	Regulating antioxidant system	Primed in melatonin for 24 h	1 μM	Promotes seed germination	[89]
Crops (Rice/ Wheat/ Maize/etc.)	Heat	Improves the water use efficiency (WUE) and nutrient homeostasis	NA	NA	Regulating crosstalk with different osmolytes and hormones	[92]
Pepper	Cold	Improving water relations and photosynthetic parameters	Soil drench	5 µM	Improve plant growth and early yield potential under chilling stress	[100]
Tomato	Cadmium	Enhanced antioxidant defense and redox homeostasis	Foliar application	100 µM	Improved osmotic balance and nutrient absorption	[107]
Radish	lead	Inducing DNA demethylation of metal transporters and antioxidant genes	Foliar application	50 µM	Reducing Pb accumulation	[111]
Soybean	Growth and development	Balancing redox homeostasis	Soil drench	100 µM	Reprogramming the biochemical metabolism	[90]
Rice	Root elongation and root growth	Regulation of melatonin biosynthesis	Medium	1 μM	Regulating both seminal root length and root growth after germination	[115]
Apple	Root formation	Inducing IAA levels and root development-related genes	Medium	1.29 μM	Promoting the function of MdWOX11	[42]
Wheat	Promotes plant growth	Upregulating the activities of N uptake and metabolism-related enzymes	Hydroponic solution	1 μM	Increasing nitrogen uptake and assimilation under nitrogen-deficient conditions	[50]

**Table 2:** Role of MEL in the mitigation of biotic stress.

Plants	Herbivores /Pathogens	Mechanism	Method of Application	Dose	Effects	Ref.
Rice	Leaffolder and brown planthopper	JA, SA, MEL, flavonoids, and lignin	N/A	N/A	Resistance to leaf-feeding and phloem-feeding herbivores	[120]
American elm	Beetle	MEL, serotonin, and JA	N/A	N/A	Resistance to beetle feeding	[114]
*Nicotiana glutinosa* and tomato	Tobacco mosaic virus (TMV)	SA and expression of pathogenesis-related (PR) genes	Soil drench	100 µM	Reduction of viral growth	[146]
Apple	Apple stem grooving virus	Not reconnoitered	Shoot proliferation medium	15 μm	Removal of virus from the infected shoot tips	[147]
*Arabidopsis thaliana*	*Pseudomonas syringae*	SA, JA, and *PR* genes	Leaf infiltration	1 μm	Resistance to *P. syringae*	[149]
Rice	*Xanthomonas oryzae* pv. *oryzae*	Cell division-related genes	N/A	N/A	Reduction in bacterial growth	[127]
Tomato	*Botrytis* *cinerea*	Activities of defense-related enzymes and MeJA content	Infiltration	50 μM	Resistance to Botrytis cinerea	[118]
Cucumber	*Fusarium oxysporum*	Phenol and flavonoids	Foliar spray	100 µM	Resistance to Fusarium wilt	[150]
Wheat	*Fusarium graminearum*	ROS and cell death	Medium	4 mM	Resistance to *F. graminearum*	[151]
Pepper	*Colletotrichum gloeosporioides*	Chitinase gene and antioxidant activity	Soil drench	100 µM	Innate immunity	[152]

## Data Availability

Not applicable.
